# Exploring mental health professionals’ practice in relation to smoke-free policy within a mental health trust: a qualitative study using the COM-B model of behaviour

**DOI:** 10.1186/s12888-019-2029-3

**Published:** 2019-02-04

**Authors:** Charlie Albert Smith, Ann McNeill, Loren Kock, Lion Shahab

**Affiliations:** 1 Department of Behavioural Science and Health, University College London, 1-19 Torrington Place, London, WC1E 6BT UK; 2 National Addiction Centre, King’s College London, 4 Windsor Walk, London, SE5 8BB UK; 3 UK Centre for Tobacco and Alcohol Studies, Nottingham, NG5 1PB UK

**Keywords:** Smoke free policy, Tobacco control, Mental health, Behaviour change, COM-B

## Abstract

**Background:**

Smoking has played a significant role in the historical culture of mental healthcare settings. Mental health professionals (MHPs) often hold dismissive attitudes regarding the importance of smoking cessation in the context of mental healthcare. In 2007, English mental health inpatient buildings were required by law to become smoke-free, and healthcare trusts have more recently begun to implement comprehensive policies (i.e. smoke-free grounds and buildings) and staff training in response to national guidance. It is therefore important to explore MHPs practice around smoking, smoking cessation, and smoke-free policy adherence. This study aimed to explore these issues by using the COM-B (capability, opportunity, motivation, behaviour) model to systematically identify barriers to, and facilitators for, MHPs addressing smoking with their patients.

**Methods:**

Five focus groups with a total of 36 MHPs were conducted between March and August 2017. MHPs were recruited from one of the largest mental health trusts in Europe. Discussions were guided by a semi-structured guide. Responses were audio recorded, transcribed and coded using thematic analysis and the COM-B framework.

**Results:**

Addressing smoking with patients was undermined by MHPs’ 1) psychological capability to recall training content, misunderstand the potential benefits of addressing patient smoking and harm reduction approaches; 2) physical opportunity in terms of time constraints, and easy accessibility of tobacco in the community; 3) social opportunity in terms of increased cultural value of tobacco following inpatient smoke-free policy implementation, and lack of support from colleagues to enforce the smoke-free policy; 4) automatic motivation, including intrinsic biases regarding patients abilities and motivations to quit, and 5) reflective motivation, including perceived job role and decision making processes related to addressing behaviours deemed more important than smoking. The main facilitating factors identified were MHPs’ having opportunity in the form of patients asking directly for support, and MHPs having access to resources such as stop smoking services and spirometers.

**Conclusion:**

Multiple barriers were identified across all key domains of the COM-B framework that undermine MHPs’ practice regarding smoking cessation. Few facilitators were identified which may have implications for future smoke-free policy and clinical practice.

## Background

Over the past two decades, smoking prevalence in the general population of the United Kingdom (UK) has decreased from 27% in the mid-90’s to around 17% in 2017 [1]. However, smoking prevalence among people with mental illness continues to be around 40% [2]. Tobacco smoking is one of the single, largest preventable causes of death worldwide [3]. People with mental health conditions smoke considerably more and have increased levels of nicotine dependency, and therefore are at greater risk of smoking-related harm [2]. Research has found that smokers with mental health conditions are at increased risk of dying prematurely compared with smokers without mental health conditions [4]. One important contributor to this persistent social inequality has been the smoking culture which has been prominent throughout mental health care systems across the world; smoking breaks were believed to be the foundation whereby therapeutic relationships were built between patient and professionals, and cigarettes were used as currency by professionals to reinforce desired patient behaviour [5–7].

Fortunately, the past decade has seen a progressive movement of change, with smoke-free policies becoming commonplace across healthcare facilities [8]. In the UK, the Health Act 2006 declared all inpatient residential settings to become smoke-free as of July 2008 [9], and soon after the National Institute of Health and Care Excellence (NICE) published guidelines for secondary care services (PH48) which encouraged smoke-free policies to be extended to outdoor areas with no exceptions [10]. These comprehensive guidelines also provided recommendations on how intensive tobacco dependence treatment should be implemented in mental health services, as well as stop smoking training for front-line staff. However, research up to now has shown that services face difficulty in their attempts to enforce smoke-free policies [9–12], with one prominent concern being health professionals’ practice regarding smoking in the mental healthcare context [13]. Findings from a mixed-methods systematic review and meta-analysis reported that Mental Health Professionals (MHPs) generally hold permissive views around smoking, as well as many misconceptions regarding mental health patients’ motivations and abilities to quit [14]. Even smoking cessation counsellors in the National Health Service (NHS), who are trained to provide specialist cessation support to highly dependent smokers, report that they generally lack the necessary knowledge and resources to do so. This is in spite of demonstrating high motivation to address smoking among those with mental illness [15]. These findings suggest that the NICE guidance is not being successfully implemented into services to support mental health patients to stop smoking.

The COM-B model of behaviour change is a comprehensive framework which is based on the assumption that behaviour is the outcome of an individual’s capability to execute it, opportunity to take part in it, and the motivation to engage in it [16]. All three conditions must be met for any given behaviour to change; capability and opportunity directly influence motivation, and motivation indirectly influences capability and opportunity through behaviour. The COM-B model is a part of the behaviour change wheel [16] that can be used to identify the barriers and facilitators to behaviour change, which in turn can be used to design and implement the most appropriate set of interventions to achieve this change. This theoretical framework has been widely applied to the behaviour of non-mental health professionals [17–19], and may prove to be useful with regard to informing smoking cessation interventions within mental healthcare settings. The present study aimed to adopt the theoretical COM-B model to understand MHPs current practice around addressing smoking with their patients (‘behaviour’) as the product of MHPs ‘capability’, ‘opportunity’, and ‘motivation’. Specifically, the purpose of this paper is to systematically identify and qualitatively explore any existing barriers which currently prevent MHPs to address smoking with their patients, using the COM-B model of behaviour. This will be further informed by the theoretical domains framework (TDF), which is a validated theoretical framework used to support intervention design [20]. To the authors’ knowledge, this is the first study of its kind to incorporate a validated behavioural framework to smoking cessation practices in mental healthcare.

## Methods

### Participants

A total of 36 MHPs employed by NHS England participated in the study. Participants were recruited from five mental health services in one of the largest mental health trusts in Europe (see Table 1). No participants refused to participate. Purposive sampling was adopted as it was crucial that participants represented the diverse multidisciplinary nature of mental health teams and services. Suitable services (i.e. mental health services within the trust) were identified by the first author (CS) using the NHS trust ‘service finder’ website, which allows filters to be used in order to search for different types of services. Managers of each suitable service were contacted via email by the research team and invited to participate. This process was ongoing until the authors had agreed that theoretical saturation had been reached, following preliminary analysis of the data. MHPs either worked exclusively within inpatient or community services, although some were not exclusive to one setting due to the nature of their profession. Participating services included one primary care community-based service (Increased Access to Psychological Treatment Services; IAPTS), one forensic inpatient service, two forensic community services, and one community mood disorder service. The target patient group of services ranged from common mental illness (e.g. depression and anxiety), to more severe mental illness (e.g. bipolar and schizophrenia) and personality disorders (e.g. antisocial and borderline).

**Table 1:** Participant characteristics

Participant characteristics (n = 36)	Mean (SD)
Age (years)	38.3 (13.8)*
Years in current role	6.9 (8.1)*
Gender	n (%)
Male	9 (25)
Female	27 (75)
Service type	n (%)
Secondary care inpatient wards	1 (20)
Secondary care community services	3 (60)
Primary care community services (IAPTS)	1 (20)
Participants in each service	n (%)
Secondary care inpatient wards	4 (11.1)
Secondary care community services	15 (41.7)
Primary care community services (IAPTS)	17 (47.2)
Professional discipline	n (%)
Occupational Therapist**	1 (2.8)
Psychiatrist	4 (11.1)
Clinical Psychologist**	4 (11.1)
Trainee Clinical Psychologist	2 (5.6)
Health Psychologist	1 (2.8)
Assistant Psychologist	1 (2.8)
Psychological Wellbeing Practitioner (PWP)	5 (13.9)
Trainee PWP	2 (5.6)
IAPTS placement student	1 (2.8)
Cognitive Behavioural Therapy (SBT) Therapist	1 (2.8)
Nurse**	6 (16.7)
Student Nurse	1 (2.8)
Social worker	3 (8.3)
Forensic Mental Health Practitioner	1 (2.8)
Support worker	1 (2.8)
Administrator/assistant	2 (5.6)
Self-reported smoking status	n (%)
Non-Smoker	17 (47.2)
Ex-Smoker	13 (36.1)
Occasional/social smoker	6 (16.7)

*Age and years in current role were not disclosed by five participants

**Includes acting service leaders (one occupational therapist, one clinical psychologist, and three nurses)

### Interview protocol and data collection

Five focus groups (one per service) were conducted by the first author (CS) and a research assistant (ZA) between March and August 2017, following a semi-structured interview guide. Participants were provided with an information sheet before participation, which outlined the aims of the study. Brief introductions took place before each focus group in order for the researchers to introduce themselves and their purpose for conducting the focus group. All focus groups were audio recorded and transcribed verbatim by the first author (CS), and a random selection were cross-checked by ZA for accuracy. Data collection ceased when CS and ZA agreed that saturation had been reached, following regular discussions of preliminary themes. The focus groups were scheduled at the convenience of the participants and were conducted in an interview room on the hospital site where the services were based. The focus groups lasted between 45 and 60 min. Notes were not taken during the focus groups and no repeat interviews were conducted.

The interview protocol was devised following a systematic review of the literature [14]. The schedule was piloted in one focus group and two interviews with inpatient MHPs, and was then refined using an iterative process. The resulting topic guide included the following themes:

- Experiences of addressing smoking with mental health patients
- Attitudes toward smoking cessation and harm reduction in the mental health context
- Attitudes and adherence to the smoke-free policy and mandatory training

### Data analysis

Data were analysed using thematic analysis [21]. In the first stage of analysis, data was uploaded onto Nvivo v.11 software [22] to facilitate data management, and then all transcripts were read repeatedly by the first author to become familiar with the whole data set. All transcripts were then reviewed and meaningful text was inductively coded by CS. Three transcripts were read and coded by the third author (LK) to assure reliability. Regular meetings between CS and LK took place whereby independent coding was compared and discussed, which was an ongoing procedure until a set of established codes were agreed upon. Once all codes were agreed, CS and LK discussed and allocated each code to the appropriate component of the COM-B model. To allow flexibility, comparisons of codes to the COM-B framework were constantly being made to assure codes that could be interpreted as being more than one function of COM-B were being allocated to the most appropriate component. Regular discussions took place during this process to assure that all codes were allocated to the most appropriate COM-B domain, and an inquiry audit was used whereby the remaining authors who were not involved with the interviews and initial coding processes monitored the research process and data analysis. Trustworthiness among authors was established through open and honest discussions, and all authors had good knowledge of the COM-B and TDF frameworks.

## Results

### Themes

#### Psychological capability: Having the knowledge and skills to address smoking with patients

Generally, MHPs were aware that smoking was a common behaviour among their patient groups. Only three MHPs stated that they had read the smoke-free policy; many misunderstood the purpose of the policy, understanding it to be for “practicality” purposes rather than to promote non-smoking and improve the health of patients and staff:

*“I didn’t understand that the policy was around helping people to change their behaviour. I just thought it was the logistics and practicalities of don’t smoke in the hospital grounds. In the same way as you wouldn’t expect people sitting out there drinking bottles of vodka. But that doesn’t necessarily mean you are there trying to change those people’s problems with alcohol. So if that is the policy then that is news to me.” (Male, ex-smoker, Clinical Psychologist, community based).[…]*

*“Even in inpatient setting, when you speak to them about smoking cessation, it’s not because of stop smoking. It’s because they are not allowed to smoke in hospitals. So you don’t prescribe something based on someone wanting to stop smoking. You prescribe to keep them on nicotine whilst they are on the ward… So you talk about it, but you prescribe it in the end just to pull them through until their next cigarette.” (Male, occasional smoker, Psychiatrist, community & inpatient based).*

The majority of MHPs indicated that they had completed the online smoke-free mandatory training. However, a common issue which was observed in all focus groups was that many MHPs could not remember the content of this training. This was expressed either indirectly (i.e. verbally questioning if particular topics were covered) or directly; the latter being attributed to the large amount of content, as well as the perceived complexity and irrelevance to clinical practice. It was common for MHPs to mention the difficulty they faced in passing the online assignment following the training:

*“I think almost too much information, and the part that’s more relevant to me was right at the end, like how you have those conversations with people, and I think if the training was just that in a more experiential way, like role plays. But it does help you practice to have those conversations with people and how to bring it up, because its uncomfortable, and that felt more relevant. But because it was right at the end, I was fed up by then. I noticed that I had that temptation to skip through it.” (Female, non-smoker, Clinical Psychologist, IAPTS). […]*

*“Practically speaking as a clinician and a manager as well, I think that it’s too long. I think there is way too much science in there for the majority of clinical staff in the trust. Hardly any clinical staff need to know about the neuro pathways that affect nicotine addiction and its interaction with antipsychotic drugs. It’s kind of overshot the mark massively. I think it’s good that we should all have some training, but I think it’s too much and pitched at the wrong level” – (Male, Ex-Smoker, Clinical Psychologist and service manager, IAPTS).*

Moreover, knowledge around alternative approaches, such as tobacco harm reduction, was generally poor among MHPs. Those who were ill-informed criticised it due to believing patients adopting this approach would take in higher concentrations of nicotine through alternative sources, such as Nicotine Replacement Therapy (NRT) and electronic cigarettes. This was often believed to be problematic, and as a result, many of these MHPs indicated that it was better for patients to continue smoking rather than adopting harm-reduction as an alternative strategy:

*“The more it goes on the more upset I am about prescribing all these extras because sometimes people use more nicotine than if they were allowed to smoke every two hours.” (Female, ex-smoker, Psychiatrist, Community & inpatient based). […].*

*“I’ve got limited knowledge, but I think realistically if you’re able to just stop, then possibly you’re going to more likely have stayed stop without having to rely on something else. Like some people get addicted to the patches and the tablets; all these different things that they are trying to do to help them stop. But it’s just transferring that addiction to another area.” (Female, ex-smoker, PWP, community based).*

#### Physical opportunity: Environmental factors that influence MHPs practice relating to addressing smoking with patients

Many MHPs indicated that they record patients’ smoking status at first point of contact with the service as this is mandatory. Additionally, they indicated that they would be willing to discuss smoking with their patients, although this was predominantly if patients provided the opportunity for discussion by directly asking for advice during sessions, and if professionals felt they had time:

*“I have talked to people who felt smoking was a major problem for them and quitting smoking was a major goal. I have done some problem solving around that in terms of if they are using it, what else can they be doing. But that’s usually if they have brought it to me with something that they need help with. As opposed to me saying let’s sit and talk about smoking.” (Female, non-smoker, PWP, IAPTS). […]*

*“If they tick on the form that actually I’m happy with the level that I’m smoking, that’s not something I’m looking to make changes in, then it feels a bit uncomfortable to bring it up in the context of having a lot of things to do in that assessment anyway.” (Female, non-smoker, clinical psychologist, IAPTS).*

However, some MHPs indicated they would take certain situations as opportunities to initiate a discussion around smoking. One manager of an inpatient ward spoke about how she takes the opportunity to discuss smoking with patients while escorting them on community leave. Yet, in spite of acknowledging that facilitating patient smoking is forbidden, she explained how being outside of the hospital perimeter makes it difficult to physically stop patients from smoking tobacco:

*“I think our time out of the ward is a very good opportunity to talk about quitting smoking, and plus the fact that when they are on escorted leave, town leave not ground leave, they are not really supposed to smoke when they are on their leave. But of course, you can’t actually physically stop them” (Female, ex-smoker, Nurse & ward manager, inpatient based).*

Similarly, almost all MHPs working in the community expressed how difficult it is to support patients to stop smoking in the community due to tobacco being widely available to community patients when they are not on hospital premises. Many MHPs provided examples of how patients continue to bring tobacco onto hospital sites and smoke in the presence of no-smoking signs:

*“The problem with enforcement I think is coming from inpatient to community. Our patients don’t give a monkey about smoking outside the door, next to the no smoking sign. Whereas I think inpatient is able to enforce it a little bit differently, particularly you know they have to go outside the grounds of the unit or whatever. But yeah it’s hard to see how it’s possible or how that problem to getting people to take it seriously. Do you man handle people off the grounds? I don’t really understand.” (Male, ex-smoker, Clinical Psychologist, community based).*

One MHP who reported having experience in both inpatient and community services described how having access to certain resources helped to engage mental health patients to consider quitting smoking. These resources included the use of a spirometer and having a stop smoking service onsite where patients could be referred to:

*“My best experience of trying to address people's smoking has been with the use of a spirometer that gives you a measurement of lung age, which in the previous service where I worked we had one… I was looking after people in their 40s and most of them had lung ages of a very heavy smoking population and a lot of them had lung ages between 80-110, and that was a really great motivational tool. The other advantage about this particular service is that there was a dedicated smoking cessation team and dedicated clinic and groups, so it was very easy to slot people in, and it was very clear who was going to see them and where they were going to go. Those people were very accessible, so it all worked really well.” (Female, ex-smoker, Psychiatrist, community & inpatient based).*

#### Social opportunity: Social factors that influence MHPs practice relating to addressing smoking with patients

Some MHPs acknowledged how smoking was once an activity shared between staff and patients, and how the trust has progressed in de-normalising this social culture which was once ingrained into the psychiatric community. However, numerous accounts of policy breaching were discussed among participants in the context of both inpatient and outpatient settings which indicated a different culture developing. Many MHPs spoke about this new culture whereby patients frequently hide tobacco around hospital grounds due to not being allowed to take tobacco onto inpatient units, which are then sometimes searched for by other patients:

*“I think one of the other things you find is they hide their cigarettes and come back to them a few hours later. I’ve seen people do that here… And it becomes like an Easter egg hunt and I think it’s because people might go and they know where their friends are hiding them. I think there has got to be more of a policy around how you handle that”. (Male, occasional smoker, Forensic Mental Health Practitioner, community based).*

Similarly, some MHPs spoke about how smoking continues to be an issue on the wards, and how the smoke-free policy has led to tobacco becoming a valuable currency among patients. As a result, some MHPs discussed how patients are now under pressure to bring tobacco and smoking-related paraphernalia onto the wards for fellow peers:

*“People are under pressure to bring cigarettes and lighters in onto the ward whereas before you didn’t have that pressure to bring cigarettes.” (Female, ex-smoker, Psychiatrist, community & inpatient based). […]*

*“Yes, and you have this double bubble called debts. So everything is one hundred percent. So if you borrow one cigarette, you may as well bring two cigarettes back” (Male, ex-smoker, Psychiatrist, community & inpatient based).*

Although inpatient policy breaches were less commonly reported compared with community services, inconsistent policy implementation among inpatient staff may jeopardise MHPs ability to successfully maintain this momentum. One MHP shared an experience of how poor support from his colleagues undermined his efforts to enforce the smoke-free policy on one inpatient unit:

*“It’s good if it gets implemented and followed through properly, but it doesn’t. Patients keep on smoking on the wards and staff are scared to confront patients, so they don’t do anything about it. I regularly go on the ward and being an ex or occasional smoker, I go on and immediately smell it, and I say ugh who’s smoking? I walk straight to the room of the patient smoking and I say this patient is smoking, and the response is, oh no he’s not! Which is ridiculous! So I don’t think it’s getting used as it should”. (Male, occasional smoker, Psychiatrist, community and inpatient based).*

#### Automatic motivation: Biases that affect MHPs practice relating to addressing smoking with patients

It was apparent that many MHPs held intrinsic biases regarding patients’ smoking behaviour. Some MHPs assumed that patients already have the necessary tools required to successfully quit smoking, and so smoking was not considered to be a topic which needs to be addressed with patients during clinical contact:

*“I think it’s common knowledge and everyone knows the consequences of smoking, so why do I have to state the obvious every time I see the clients, because they are not stupid. They’ve got the knowledge but they made the choice they want to make.” (Female, occasional smoker, Nurse, community based).*

More often, MHPs commented on patients’ abilities and motivation to quit smoking. Specifically, many believed that mental health patients are resistant to address their smoking, as tobacco was considered to be one of the few pleasures these patients have in their lives:

*“From experience I have not really met many who would ever want to give up. That seems to be the one thing they like about their life.” (Male, ex-smoker, Clinical Psychologist, community based). […].*

*“I think overall they are locked up and banged up in a secure environment, so what can they do? So obviously when they go on their escorted or unescorted leave, invariably they do smoke.” (Female, ex-smoker, Nurse & Ward Manager, inpatient based).*

Some MHPs commented on how patients do not perceive smoking as being a psychiatric problem which should be addressed within a mental health service. Subsequently, fears of inflicting misbalance to the power dynamic of the established therapeutic relationship may subconsciously deter professionals from advising patients to quit smoking:

*“They can’t see it as a psychiatric problem, so they can’t see why you’re asking. It’s none of your business kind of thing.” (Male, ex-smoker, Psychiatrist, community & inpatient based). […]*

*“Some patients interpret it as part of control. Because of the dynamics it’s why are you telling me what to do. Can I tell you to stop smoking? It’s my life…” (Male, non-smoker, Social Worker, community Based).*

#### Reflective motivation: Self-conscious decision making and reasoning that influence MHPs practice relating to addressing smoking with patients

The extent to which MHPs felt addressing smoking was part of their role varied. Within community settings, some MHPs expressed how their role is fundamentally to provide information and raise awareness, with few mentioning they would take it upon themselves to provide smoking cessation advice:

“*We have got a responsibility to provide them with some information about health promotion and smoking comes into that. But I think in the community that’s probably where our role ends. You know, we can offer them where to find some more information or give some advice, but Erm… yeah. I think that’s it really”. (Female, Occasional Smoker, Nurse & Team leader, Community based).*

Other professionals within the community expressed their views of how the trust’s requirement to monitor patients’ smoking was something which had been imposed on them, rather than it being something which they would pursue otherwise:

*“I think that it has been made a part of my job, more than I would do it as part of my job, with the smoking. It’s an unhealthy habit, there are lots of unhealthy habits. But its more something I’ve kind of been told to do.” (Female, ex-smoker, Social Worker, community based).*

Explanations given as to why smoking was not necessarily a priority for MHPs in the community were often based around the complexity and heterogeneity of mental health; often being associated with many complex needs and behaviours which MHPs place greater emphasis on:

*“I don’t think I’ve ever offered. It just never comes to mind. I don’t know why. But maybe the number of problems people tend to come to me with, that doesn’t seem to ever factor in their list of… well it’s not even a problem for them, but even if it is then its way down the list behind other drugs or other problems that they are having.” (Male, ex-smoker, Clinical Psychologist, community based).*

However, although many MHPs placed greater emphasis on other areas of the patient’s health, it was common for some MHPs to reflect on their practice and state that they would address smoking with patients in the context of there being a clear physical health or financial issue:

*“I think sometimes because people have got issues with their medication as well, if they are mixing the two together, you might be more likely to focus on discussing their issues about their medication with them and that you can’t mix two difficult issues together and you’re unlikely to be successful in discussing both so unless there is an obvious physical health problem that was becoming a major concern, I would probably choose the medication.”(Male, ex-smoker, Social Worker, community based).* […]

*“I often ask about it if they are a smoker and just say, is that something you’re looking to give up or not? Especially if there are financial issues or health issues or maybe if they are smoking a lot of marijuana or something like that.” (Female, non-smoker, Clinical Psychologist, IAPTS).*

Perhaps due to the greater emphasis placed on smoking and inpatient settings, these MHPs felt more obliged to stopping patients from smoking, which was also often noted by MHPs in the community. However, although it was their responsibility to enforce policies and raise awareness about smoking, professionals working on the wards expressed how it was not their responsibility to “police” smoking. As a result, some were often complacent with patients’ smoking outside the premises, and in some instances condoned limited smoking when patients were granted leave in the context of harm-reduction:

*“It isn’t our role as clinicians to police smoking. If it were illegal which cannabis is, we take a different approach on that obviously. But with smoking as of yet not illegal, we can’t stop them. Literally. When they go out on their leave, which isn’t every minute of every day, they may smoke. But while they are in the building, they are not smoking.” (Female, ex-smoker, Nurse & Ward Manager, inpatient based).* […]

*“I don’t allow them to smoke that much so they are only allowed two cigarettes when they go out on leave. It causes a lot of problems with negotiations.” (Female, ex-smoker, Psychiatrist, community & inpatient based).*

Many MHPs in community services stated that they would not approach patients who were smoking on hospital grounds if they did not know them. A common reason for this was fear of experiencing potential conflict:

*“In all honesty, I’d worry about being thumped! You don’t know what the patient’s history is. You don’t know what the risk history is. I think that’s tricky.” (Female, ex-smoker, Psychiatrist, community & inpatient based). […]*

*“I’ve seen people smoking, just near our office actually, and I feel a bit conflicted and there is some dissonance. Because I think it probably is my role as a senior clinician to enforce the policy, it’s probably all of our role to do that really. But at the same time I have other conflicting ideas which get in the way of me doing that. Like… I don’t want to get into conflict with someone and they are not a patient being seen in our team, they are clearly inpatients. And… I’m really busy and I’ve got other things I could be doing with my time, so there are always excuses that I think I could make for myself to not get into a conflict situation about it” (Male, Ex-Smoker, Clinical Psychologist and service manager, IAPTS).*

## Discussion

Findings from this study suggest that addressing smoking with mental health patients may be undermined by MHPs’ capability (knowledge and memory); opportunity (environmental influences and peer support); and motivation (biases, self-identity and priorities). Particular barriers included misunderstanding of nicotine and harm reduction, unfamiliarity with the smoke-free policy, inability to recall training content; time constraints, resilient smoking culture, intrinsic biases, perceived job role and responsibilities/priorities. A key facilitator to MHPs addressing smoking with patients was MHPs opportunity to do so. Specifically, addressing smoking with patients was facilitated by patients bringing smoking as an agenda to meetings with MHPs, as well as MHPs having access to resources such as on-site stop smoking services and spirometers. The authors discuss these findings below, and provide a number of potential recommendations for future research and clinical services which have been informed from the wider literature and the behaviour change wheel [16]. Further implications and recommendations are listed in Table 2.

**Table 2:** Identified barriers and suggested interventions that have been informed by the literature, COM-B and TDF [16]

Barriers identified	COM-B	TDF	Intervention function	Possible intervention
Poor comprehension of harm reduction approaches, smoke-free policy and its purpose. Unable to recall training content MHPs lack the confidence to address smoking with patients who do not initially indicate willingness to quit.	Psychological capability	Knowledge Memory, attention and decision Cognitive and interpersonal skills	Education Training Environmental restructuring Enablement	Brief, face-to-face or online smoking cessation training tailored specifically to MHPs roles [35]. Training which allows MHPs to practice the skills required to address smoking with patients (e.g. role play group training), thus improving capability as well as confidence and motivation [24]
Inability to monitor patients’ smoking in community services. Lack of time and resources to provide smoking cessation interventions (only ask about smoking at initial assessment).	Physical opportunity	Environmental context and resources	Training Restriction Environmental restructuring Enablement	Improve access to specialist equipment, such as spirometers and CO monitors [26] [27]. Improve communications/referral process between mental healthcare (non-smoking specialists) services and stop smoking services (smoking specialists) [36].
Tobacco has become a prominent contraband item in the community and inpatient settings. Lack of support from colleagues to enforce smoke-free policy.	Social opportunity	Social influences	Restriction Environmental restructuring Modelling Enablement	Group training to encourage team work and shared learning across different care teams [24]. Videos of positive attitudes of fellow healthcare providers and colleagues. Increased vigilance to prevent anti-social behaviour (i.e. hiding tobacco in bushes and units) that is sensitive to mental healthcare contexts (e.g. ‘watchful eyes’ intervention [37] has been found to be effective in other contexts, but may not be appropriate in settings whereby patients experience paranoia).
Intrinsic biases regarding mental health and smoking.	Automatic motivation	Reinforcement Emotion	Training Incentivisation Persuasion Coercion	Improve clinical reasoning and decision making skills, such as through reflective practice and active metacognitive review [23]. Use of emotive videos of patients who want to quit, but cannot due to their mental health.
Smoke-free policy and training lacks relevance to non-inpatient services. Prioritise alcohol and other substances over tobacco. Only intervene with tobacco use in light of financial or physical health issue. Belief that addressing smoking with patients could provoke retaliation from some patients.	Reflective motivation	Professional role and identity Beliefs about capabilities Beliefs about consequences Intentions Goals	Education Persuasion Modelling Incentivisation Coercion Enablement	Tailoring training to clinical setting/role, and manuals to aid MHPs [23]. Incorporate smoking cessation into other treatments [34]. Improving awareness that ‘preventing’ is more beneficial compared with ‘treating’. Improved dissemination of findings that show violence has decreased in inpatient setting following smoke-free policy implementation [31].

Regarding capability, it was found that many MHPs hold misconceptions in relation to patients’ abilities to quit smoking, which reflect the views found by the meta-analysis of Sheals and colleagues [14]. Moreover, MHPs tend to believe that alternative approaches to cessation, such as harm reduction, have little or no benefit to these patients due to misinterpreting nicotine as being the harmful substance where abstinence is needed [6]. This is despite many of these MHPs claiming to have engaged in mandatory online smoking cessation training. One notable finding of the present study was that MHPs in community settings could not recall the content of this training, with many stating that the test assignment at the end was difficult to pass. Moreover, many professionals commented on the small applicability they felt the smoke-free policy and training had to non-inpatient services, which was often provided as the reason why many had not read the smoke-free policy. It may be that the rationale for the policy and need for training has not been communicated clearly to professionals, which is a recommendation in the NICE guidance [10]. Tailoring training content to different professional backgrounds and clinical settings, as well as incorporating accessible resources such as professional manuals, could improve knowledge and bridge the existing gap between training targets and current practice, especially in community mental health services [23]. In addition, it is noteworthy that participants who had completed training only engaged with the mandatory online smoking cessation training, and had not participated in the advanced optional face-to-face training which is provided by the trust. Research suggests that group training is more effective than web-enabled video training with regard to improving outcomes that are affected by the availability of professional knowledge [24]. Indeed, a number of MHPs commented on how they felt uncomfortable to bring up smoking cessation with their patients, and so the incorporation of group-based training may improve MHP's communication skills and confidence through activities such as role-play and feedback [24]. Making these changes to the resources available to MHPs may therefore improve their capability which could have positive implications for MHP's practice, as well as their motivation to address smoking with their patients. As this study did not include professionals who had received the advanced optional training, further research should investigate whether these barriers relating to capability of the COM-B model differ in this group, as this may also provide useful when developing the mandatory training.

Regarding opportunity, it is apparent that the environmental infrastructure of services affects MHP's ability to address smoking with patients and enforce the smoke free policy. MHPs in inpatient settings have increased daily contact with patients compared with MHPs who solely practice in the community, and so have more physical opportunities to address smoking with patients. This study highlighted the lack of physical opportunity which community MHPs feel they have to discuss smoking with their patients; often referring to time constraints and the inability to monitor patients’ progress because of having limited face-to-face contact with patients. In line with the behaviour change wheel, enabling MHPs by increasing access to resources which can be used in the community may assist in overcoming this barrier, which could improve the motivation of both the patient and MHPs. One participant of the present study mentioned how the close proximity and easy access of a stop smoking service at one hospital aided her in supporting patients to quit smoking. However, recent funding budget cuts across English NHS services have had a negative impact on stop smoking services [25], resulting in some locations not having easy access to this resource. Nonetheless, the same MHP also spoke of her experience using a spirometer which helped her in the past to engage patients in discussion regarding their smoking. One randomised controlled trial (RCT) found that telling smokers their lung age significantly improved the likelihood of them quitting smoking at 12 month follow-up [26]. Therefore, increasing accessibility to spirometers in the community may facilitate the interaction between MHPs and patients with regard to smoking cessation, and may therefore resolve one existing problem community MHPs face in monitoring progress. Increasing access to such resources may be even more crucial for disadvantaged services in which there is no on-site stop smoking service. This was the case for a number of services recruited for the present study, which further demonstrates how NICE recommendations are not currently being met [10]. Furthermore, as patients’ directly requesting help from MHPs was a facilitating factor that encouraged them to support patients, it is feasible that the inclusion of spirometers during routine smoking screenings would potentially motivate more patients to quit and seek support, which in turn would encourage MHPs to provide this requested support. Another suggestion may also be to provide services with carbon monoxide testing machines to allow MHPs to record and monitor patients’ smoking behaviour, which is a NICE recommendation for identifying pregnant women who smoke [27]. Indeed, some studies have supported the use of exhaled carbon monoxide readings for encouraging quit attempts and cessation [28, 29]. Further research should explore the potential impact of such measures becoming an essential component of smoking status screenings within mental health services.

Regarding motivation, it was apparent that many MHPs held intrinsic biases regarding patients’ abilities and desires to address their patient’s smoking. This is in line with findings of a systematic review [14]. In particular, community MHPs were often permissive about smoking; believing that addressing smoking was not their role, and prioritising other behaviours such as illicit substance use, which were deemed more important to this patient group. Consequently, smoking is often only asked in the first initial assessment and rarely followed up in the absence of physical and financial burdens; both being inevitable as smoking has been found to be an prominent cause of social inequality in terms of health and poverty for this population [30]. Again, this is not in accordance with the NICE guidance that recommends continuity of support from inpatient to community services [10]. One identified barrier was that many MHPs in the community questioned the relevance of the smoking cessation training to their daily clinical work. As a result, one Clinical Psychologist based in an IAPTS service mentioned how her motivation to complete the training diminished; causing temptation to skip content which she perceived to be relevant to her practice. It may be that training developers should consider more practical domains in future training development in order to have the highest impact. This would particularly be the case for MHPs in the community who often stated that they would not approach a patient smoking on hospital grounds due to time constraints and also fearing violence in retaliation. Research has reported that an increase in violence was a major concern for inpatient MHPs before the implementation of smoke-free policies, although the opposite was found in reality [31]. Therefore, improving education and communication of these findings, along with offering practical workshops where MHPs can practice instigating conversations with patients regarding smoking, may succeed in improving MHPs confidence to address smoking more frequently and seriously with their patients. The potential to achieve reductions in violent incidences as a result of supporting patients to quit smoking may particularly be an incentive for forensic services where general risk of violence may be higher [32].

Finally, MHPs tended to believe that mental health patients have multiple behaviours that take priority over smoking, such as illicit substance misuse. These findings are in line with those reported in a recent qualitative study; patients of substance misuse services are motivated to apply their learning to smoking cessation but do not receive support or encouragement from staff within the substance misuse services [33]. Research has shown that smoking cessation can be successfully incorporated into treatments addressing illicit substance use without jeopardising recovery goals, which contradicts these arguments made by some of the participants in the present study [34]. In line with the behaviour change wheel, raising awareness of this, in addition to emphasising the degree of impact which tobacco smoking has on physical and mental wellbeing, may encourage MHPs to consider incorporating smoking cessation in their practice when addressing illicit substance misuse with patients. Training developers should consider incorporating content regarding this common barrier in future training programmes.

## Limitations

The purposeful sampling methods to recruit MHPs from only five settings within one mental health trust in England means the findings cannot be generalised to the MHP population, or other settings within that trust or other mental health trusts. Given the multidisciplinary nature of mental health services and the small number of participants in this study, the sample is also not representative of the services they work in. Moreover, due to the design of the study, as well as the complexity of the healthcare pathway, systematic subgroup comparisons were not possible. Indeed, many professionals, such as psychologists and psychiatrists, work in both inpatient and community services, thus making it difficult to classify these MHPs as community or inpatient based. This also made systematic comparisons between inpatient and community MHPs difficult. However, this study highlighted a number of differences between inpatient and community services which have implications for the development of smoke-free policy and smoking cessation practice in mental healthcare pathways. One comparison of interest was whether smoking or vaping status influenced MHPs responses, which this study found to not be the case. Nonetheless, further research should explore potential relationships such as these more systematically.

The application of COM-B allowed us to identify factors that impacted MHPs practice regarding smoking in their roles. However, we encountered some difficulty in separating the COM-B categories. For instance, it was difficult to distinguish whether inability to recall training content was the result of MHPs memory and attention (i.e. psychological capability) or their lack of concern to remember (i.e. motivation). Where possible, we used author agreement to provide a parsimonious analysis of responses to fit the COM-B framework. As the interview guide was not based on the COM-B model, the data allowed for inductive coding which could be classified into a COM-B framework, therefore not limiting the scope of the data.

Finally, the role of the researcher (CS) should be considered when interpreting the data of the present study. Although the researcher had attended a qualitative training course prior to the focus groups, it is possible that previous experiences and beliefs could have influenced the study. Unfortunately, it was not practical for participants to comment on the transcripts before data analysis begun, which would have further enhanced trustworthiness and reduced the potential impact of researcher bias. Moreover, the researcher’s previous clinical experience of working in one of the services recruited in the present study could have influenced how data was interpreted and coded. Attempts to minimise this included use of a semi-structured interview guide, as well as a second coder (LK), who had no prior relationship with participants. It is also feasible that having an established working relationship prior to interviews could have influenced participants’ responses (e.g. social desirability bias). However, establishing data saturation through identifying common themes across focus groups suggests this would have had minimal impact to the main findings.

## Conclusions

To our knowledge, this is the first study that has applied a comprehensive model of behaviour to the exploration of MHPs practice regarding smoking cessation across a range of mental healthcare settings. In spite of MHPs having specified duties outlined in the smoke-free policy, as well as being in receipt of training, many continue to hold misconceptions about their patients’ abilities to stop smoking, as well as the potential benefits that could be achieved through supporting them to do so. This unawareness in combination with environmental and resource constraints negatively affects community MHPs motivation to address smoking with their patients.

## Abbreviations

COM-B: Capability, opportunity, motivation – behaviour model
MHP: Mental health professionals
NHS: National health service
NICE: National Institute for Health and Care Excellence
TDF: Theoretical domain framework
